# Muscular Function as an Alternative to Identify Cognitive Impairment: A Secondary Analysis From SABE Colombia

**DOI:** 10.3389/fneur.2022.695253

**Published:** 2022-02-18

**Authors:** Elkin Garcia-Cifuentes, Felipe Botero-Rodríguez, Felipe Ramirez Velandia, Angela Iragorri, Isabel Marquez, Geronimo Gelvis-Ortiz, María-Fernanda Acosta, Alberto Jaramillo-Jimenez, Francisco Lopera, Carlos Alberto Cano-Gutiérrez

**Affiliations:** ^1^Facultad de Medicina, Semillero de Neurociencias y Envejecimiento, Instituto de Envejecimiento, Pontificia Universidad Javeriana, Bogotá, Colombia; ^2^Unidad de Neurología, Hospital Universitario San Ignacio, Bogotá, Colombia; ^3^Departamento de Epidemiologia Clínica y Bioestadística, Pontificia Universidad Javeriana, Bogotá, Colombia; ^4^Facultad de Medicina, Grupo Neurociencias de Antioquia, Universidad de Antioquia, Medellín, Colombia; ^5^Centre for Age-Related Medicine (SESAM), Stavanger University Hospital, Stavanger, Norway; ^6^Faculty of Health Sciences, University of Stavanger, Stavanger, Norway; ^7^Facultad de Medicina, Grupo Neuropsicología y Conducta, Universidad de Antioquia, Medellín, Colombia; ^8^Facultad de Medicina, Semillero de Investigación SINAPSIS, Universidad de Antioquia, Medellín, Colombia; ^9^Unidad de Geriatría, Hospital Universitario San Ignacio, Bogotá, Colombia

**Keywords:** gait speed, handgrip strength, cognitive impairment, biomarker, pre-clinical dementia, motor dysfunction

## Abstract

**Background:**

Identification of cognitive impairment is based traditionally on the neuropsychological tests and biomarkers that are not available widely. This study aimed to establish the association between motor function (gait speed and handgrip strength) and cognitive performance in the Mini-Mental State Examination, globally and by domains. A secondary goal was calculating a cut-off point for gait speed and handgrip strength to classify older adults as cognitively impaired.

**Methods:**

This is a secondary analysis of SABE Colombia (Salud, Bienestar & Envejecimiento), a survey that was conducted in 2015 on health, wellbeing, and aging in Colombia. This study used linear regression models to search for an association between motor function and cognitive performance. The accuracy of motor function measurements in identifying cognitive impairment was assessed with receiver operating characteristic (ROC) curves. This study also analyzed other clinical and sociodemographical variables.

**Results:**

Gait speed was associated with orientation (*r*^2^ = 0.16), language (*r*^2^ = 0.15), recall memory (*r*^2^ = 0.14), and counting (*r*^2^ = 0.08). Similarly, handgrip strength was associated with orientation (*r*^2^ = 0.175), language (*r*^2^ = 0.164), recall memory (*r*^2^ = 0.137), and counting (*r*^2^ = 0.08). To differentiate older adults with and without cognitive impairment, a gait speed cut-off point of 0.59 m/s had an area under the curve (AUC) of 0.629 (0.613–0.646), and a weak handgrip (strength below 17.5 kg) had an AUC of 0.653 (0.645-0.661). The cut-off points for handgrip strength and gait speed were significantly higher in male participants.

**Conclusions:**

Gait speed and handgrip strength are similarly associated with the cognitive performance, exhibiting the most extensive association with orientation and language domains of the Mini-Mental State Examination. Gait speed and handgrip strength can easily be measured by any clinician, and they prove to be useful screening tools to detect cognitive impairment.

## Introduction

Dementia has become a worldwide health priority that affects the quality of life of older adults and their caregivers (1). In Colombia, identification of cognitive impairment has been traditionally based on neuropsychological tests, imaging, and molecular biomarkers that are not widely available. This poses a major challenge in the timely diagnosis of dementia. The use of non-cognitive biomarkers is an emerging approach for early diagnosis of cognitive impairment (2) and, consequently, for setting up preventive strategies for dementia in low- and middle-income countries such as Colombia (3).

Growing evidence suggests that dementia, particularly Alzheimer's Disease (AD), is a *continuum* with a long pre-clinical stage that may present with early motor symptoms (4–6). In recent years, identification of the association between cognitive and motor performance suggests that these functions share neural networks in the frontal-hippocampal cortex. Impairment of this neural network can manifest as a concurrent decline of the motor and cognitive functions (7). This means motor function assessment can be a useful correlate with cognition and a promising predictor of mild cognitive impairment (MCI) and dementia (2).

In Colombia, prior reports demonstrate an association between motor dysfunction and dementia (8, 9). In addition, the 5th Canadian Consensus Conference suggested that individuals with subjective memory complaints and motor dysfunction (i.e., gait speed disturbances, and dual-task gait impairment) are prone to developing cognitive decline and should undergo close follow-up and screening (10). However, currently in the Latin America, no standardized screening protocols for cognitive impairment use motor function measurements.

Handgrip strength (HS) assessed with a hand-held dynamometer is proven as a good indicator of the whole muscular strength and wellbeing in the older adults. The HS is influenced by factors such as age, sex, socioeconomic status, and level of physical activity (11). Several reports in the literature suggest that HS might be a good predictor for the risk of cognitive decline (8), chronic diseases, depression, frailty, and dependency in instrumental activities of daily living (IADL) (12, 13).

Gait speed (GS) is another muscular function associated with cognitive function (14, 15) since it integrates motor, perceptual, and cognitive processes. Abnormalities in GS precede cognitive decline by the several years (16–18). A recent publication considered GS measurement as a novel biomarker of cognitive decline, MCI, AD, and other dementias (19).

Measurements of HS and GS are easy to perform, objective, non-invasive, low cost, widely available, and safe. Those characteristics make them acceptable, easy to generalize, and valuable in the clinical assessment of older adults. There is limited knowledge, however, about the cognitive domains predominantly related to motor performance.

This study aimed to establish the association between motor function, assessed with GS and HS, and cognitive function, assessed with the Mini-Mental State Examination (MMSE). A secondary goal was to determine a cut-off point for GS and HS and the accuracy of these motor tests in the identification of cognitive impairment. Based on preliminary reports (8, 9), it can be hypothesized that reduced motor function might be associated with global cognition in the MMSE and some specific domains, such as orientation and memory.

## Materials and Methods

### Study Design

This is a secondary analysis of SABE Colombia (Salud, Bienestar, & Envejecimiento) a population-based, cross-sectional study of health, wellbeing, and aging that was conducted in Colombia in 2015. The SABE Colombia included a representative sample of the Colombian population −23,694 non-institutionalized adults aged 60 years or older. The probability sampling method was clustered, multistage stratified by urban and rural areas. Methods and procedures conducted in SABE Colombia were based on those used in the international multicenter SABE study to obtain comparability, generalizability, and harmonized protocols (20) adapted to the Colombian population. The information was integrated within the general framework of the Colombian National Surveys System. Other technical details of the SABE Colombia study can be found in the official website of the Colombian Ministry of Health and Social Protection, and other independent publications (21–23).

### Inclusion and Exclusion Criteria

**Figure 1:**
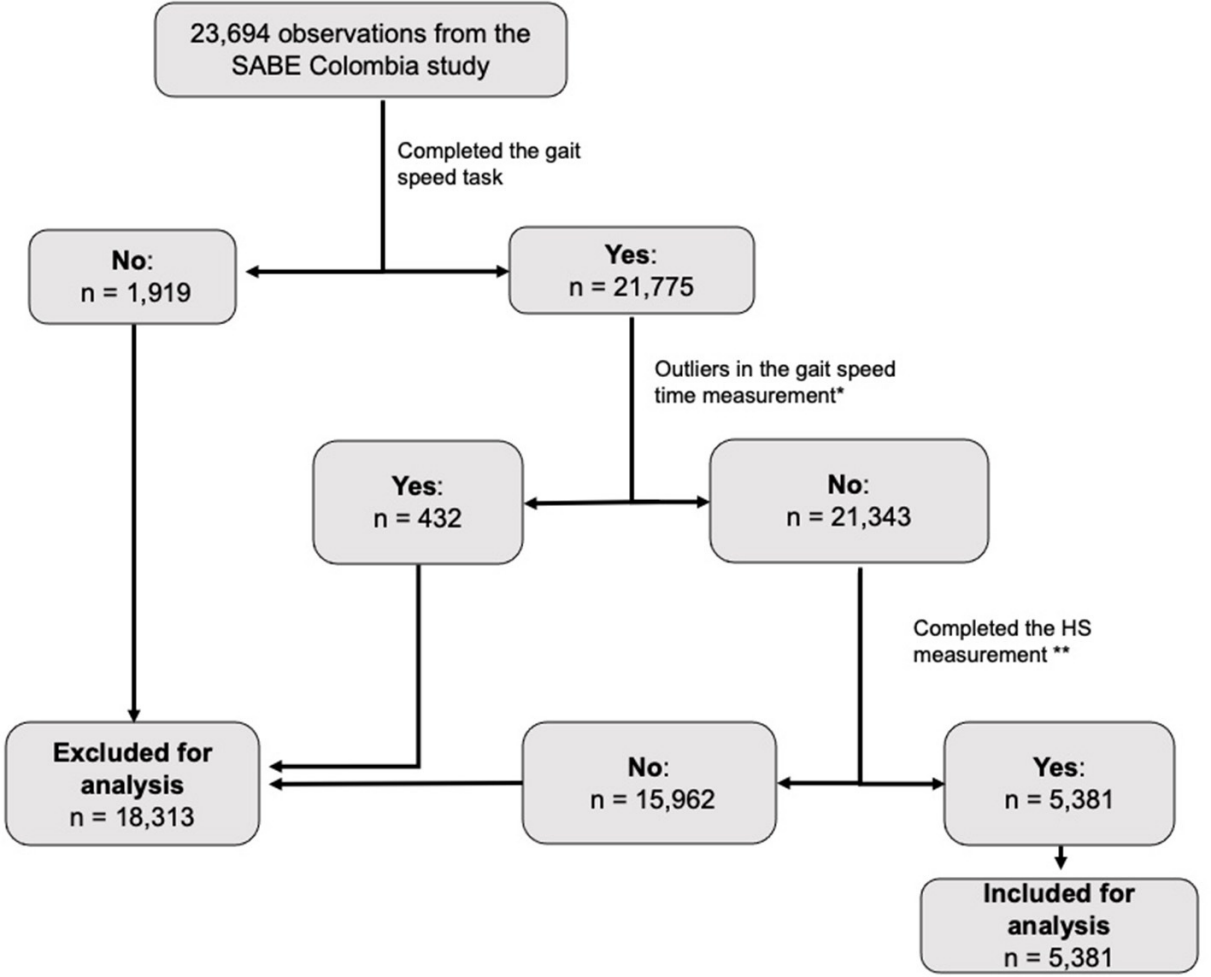
Flow-chart of the exclusion process before the statistical analysis. *Values below the p1 and above the p99 on the time spent wqalking 3 m were excluded from the analysis. **HS, Handgrip strenghth (only a subsample were indicated to use the an adjustable digital handgrip dynamometer).

The present analysis took a subsample of 5,381 SABE Colombia participants who were able to complete the GS and HS measurements. For the GS test, this study excluded outliers, who were participants with GS values below the percentile 1 or above percentile 99, as these could have been individuals who ran during the test or whose data were entered incorrectly by an examiner. Figure 1 shows a detailed flowchart with eligibility criteria and the selection of the subsample for this study.

### Variables

The dependent variables assessed in this study were the cognitive variables: the MMSE and its 4 domains (orientation, recall, counting, and language). The independent variables were the HS and GS (muscular function measurements). The possible confounders were sociodemographics (age and gender), functionality measures (Barthel and Lawton scores), comorbidities (high blood pressure, diabetes, myocardial infarction, stroke, arthropathies, and mental diseases), and anthropometrics (body mass index).

Hand grip strength: The HS was measured with an adjustable digital handgrip dynamometer (Takei Scientific Instruments Co., Tokyo, Japan). An examiner instructed each participant in the use of the dynamometer and recorded in kilograms (kg) the best score for each hand. Calculated HS was the average of the left and right hands (24).

Gait speed: The GS was computed from a subtest of the Short Physical Performance Battery (SPPB), validated for the Colombian population, and applied in SABE Colombia (19). Participants were asked to walk 3 meters at their regular pace two times from a standing position. The GS was the best time of the two trials (9).

Functional status: The study assessed basic activities of daily living with the Barthel index (from 0 to 100). Lower scores indicated a functional dependency (25). The Lawton and Brody scale (from 0 to 54), scored instrumental activities of daily living, with higher scores signaling functional impairment (26, 27).

Comorbidities: The study presented the frequency of self-reported comorbidities that had been diagnosed by a physician, including high blood pressure, myocardial infarction, stroke, diabetes, arthropathies (including arthrosis, arthritis, and rheumatoid arthritis), and mental disease.

Cognitive performance: The MMSE was used to classify individuals as cognitively impaired. The MMSE has optimal psychometric characteristics, with 88.3% sensibility and 87% specificity. There is wide support for the use of the MMSE in the initial assessment and follow-up of memory, language, orientation, and visuo-constructional capacity in people with neurocognitive disorders. The MMSE total score ranges from 0 to 30, with low values indicating worse cognitive performance (28, 29). This study used an MMSE score cutoff of ≤24 to classify individuals as cognitively impaired (30, 31).

### Statistical Analysis

This study performed a descriptive analysis of the subsample, given the quantitative nature of the variables. Central tendency and dispersion measures were calculated with the program *R* (32). The Kolmogorov-Smirnov (Lilliefors's correction) test served to determine the normal distribution of each variable.

### Assessment of the Association Among Muscular Function and Cognitive Variables

The study used various linear regression models with the cognitive variables (the MMSE total score and by domains) as dependent variables, and the other variables of interest as independent variables. The *p-*value coefficients and McFadden's R-squared were used to test the models. For the analysis of the relationship among variables, three different regression models were tested. The first *model* included GS, age, and gender as the independent variables, with the cognitive variables (the MMSE total score and by domains) as dependent variables. The second *model* included HS, age, and gender as independent variables, and the MMSE domains as the dependent variables. The *third and final model* included GS and HS, comorbidities, functionality, and sociodemographic variables as independent variables, and the MMSE domains as the dependent variables. In all these models collinearity among independent variables was ruled out by calculating the Spearman correlation coefficient, where no pair of variables had a high correlation; with Rho values ranging between −0.58 and 0.36. Also, possible interactions (or confounding effects among them) were evaluated. The independent variables in the final model were selected using backward elimination.

### Muscle Function for Assessment of Cognitive Impairment

To assess GS and HS performance in the identification of cognitive impairment, the authors, for each variable (GS and HS independently), applied the Youden index, identified optimal cut-off points with receiver operating characteristics (ROC) curve analyses, and calculated the areas under the curve (AUC). Considering that gender may affect motor performance, the authors defined different cutoff points stratified by gender with a *post-hoc* analysis, running a new ROC curve analysis for each motor function.

## Results

The SABE Colombia cohort included 23,694 older adults. Cognitive impairment was identified in 4,690 individuals, for an overall prevalence of 19.79%. In the group with cognitive impairment, 62.3% (*N* = 2,922) were females and 37.7% (*N* = 1,768) were males. The median age for this group was 77 years (IQR 14), 9 years older than individuals with normal cognition (median 68, IQR 11). In addition, the body mass index (BMI) was lower in the cognitively impaired group (median 24.64 IQR 6.59 vs. median 26.39, IQR 6.23).

Table 1 summarizes the descriptive analysis of the subsample (*N* = 5,831) by cognitive status. Out of the 5,381 participants, 4,035 had normal cognition and 1,346 had cognitive impairment. The median age was 68 years for individuals with normal cognition and 75 years for individuals with cognitive impairment. The education median was 4 and 1 year, respectively. In the group with normal cognition, 57.62% were females and 42.38% were males. In the group with cognitive impairment, 62.78% were females and 37.22% were males. In terms of muscular function, HS and GS were lower in the group with cognitive impairment.

**Table 1:** Descriptive analysis by cognitive status.

		**Total (*n* = 5,381)**	**Normal cognition, *n* = 4,035 (74.99%)**	**Cognitive impairment, *n* = 1,346 (25.01%)**
**Variable**		***n*** **(%)**		
Gender
	Female	3,180 (59.1%)	2,325 (57.62%)	845 (62.78%)
	Male	2,201 (40.9%)	1,710 (42.38%)	501 (37.22%)
Comorbidities	
	Hypertension	3,027	2,184 (54.21%)	843 (62.17%)
	Myocardial infraction	778	550 (13.64%)	228 (16.79%)
	Stroke	238	149 (3.7%)	89 (6.55%)
	Diabetes	897	657 (16.34%)	240 (17.69%)
	Arthropathies^*^	1,513	1170 (29.05%)	343 (25.33%)
	Mental Diseases^**^	485	326 (8.10%)	159 (11.76%)
			**Median (IQR)**	
Demographics	
	Age (years)		68 (10)	75 (14)
	Schooling (years)		4 (4)	1 (3)
Anthropometrics	
	Body mass index (BMI)		26.39 (6.23)	24.64 (6.59)
Muscular function	
	Hand grip strenght (kg)^$^		23.18 (8.84)	18.74 (8.7)
	Gait speed (m/s)^$^		0.723 (0.23)	0.586 (0.23)
Functionality	
	Lawton total		0 (2)	4 (10)
	Barthel index		100 (0)	100 (5)

* *Includes arthrosis, arthritis and rheumatoid arthritis*.

** *Major mental disorders*.

$ *Mean (SD) t-test*.

Table 2 summarizes the demographic-adjusted linear regression models to predict cognitive variables. The GS was associated with orientation (*r*^2^ = 0.16), language (*r*^2^ = 0.15), recall memory (*r*^2^ = 0.14), and counting (*r*^2^ = 0.08). Similarly, HS was associated with orientation (*r*^2^ = 0.175), language (*r*^2^ = 0.164), recall memory (*r*^2^ = 0.137), and counting (*r*^2^ = 0.08). Table 3 shows the final fully adjusted model exploring associations among motor function and cognitive variables. This model included covariates such as age, presence of mental disorder (the only comorbidity selected after backward elimination), and functionality with its interactions. The analysis revealed that 29.1% of the variability in orientation is attributed to the described model. Also, the coefficient in this model was large for language (*r*^2^ = 0.273), medium for memory recall (*r*^2^ = 0.193), and small for counting (*r*^2^ = 0.109).

**Table 2:** Association between motor function and cognitive domains.

**Measurement**	**Mini mental state examination**									
	**Orientation**		**Recall**		**Counting**		**Language**		**Total**	
	**R2**	***p*-value**	**R2**	***p*-value**	**R2**	***p*-value**	**R2**	***p*-value**	**R2**	***p*-value**
Gait speed^*^	0.16	<0.001	0.14	<0.001	0.08	<0.001	0.15	<0.001	0.18	<0.001
Handgrip strength^*^	0.175	<0.001	0.137	<0.001	0.08	<0.001	0.164	<0.001	0.19	<0.001

* *Both measures presented significat interaction with age*.

**Table 3:** Association's model for cognitive variables.

**Measure**	**Mini mental state examination**									
	**Orientation**		**Recall**		**Counting**		**Language**		**Total**	
	**R2**		**R2**		**R2**		**R2**		**R2**	
	0.291		0.193		0.109		0.273		0.303	
	**β (SE)**	***p*-value**	**β (SE)**	***p*-value**	**β (SE)**	***p*-value**	**β (SE)**	***p*-value**	**β (SE)**	***p*-value**
Gait speed	0.66 (0.16)	<0.001	0.29 (0.07)	<0.001	0.33 (0.11)	0.002	0.46 (0.13)	0.003	1.73 (0.38)	<0.001
Handgrip Strength	−0.14 (0.03)	<0.001	−0.04 (0.02)	0.03	−0.04 (0.02)	0.107	−0.09 (0.3)	0.37	−0.3 (0.09)	<0.001
Age	0.15 (0.09)	0.07	0.07 (0.04)	0.06	0.14 (0.05)	0.03	0.06 (0.07)	0.14	0.43 (0.21)	0.044
Mental disease	−0.16 (0.12)	0.17	−0.16 (0.05)	0.027	−0.03 (0.08)	0.74	−0.14 (0.09)	0.068	−0.44 (0.28)	0.118
Barthel	0.19 (0.07)	0.005	0.08 (0.03)	0.006	0.14 (0.05)	0.004	0.1 (0.05)	<0.001	0.51 (0.16)	0.001
Lawton	0.3 (0.08)	<0.001	0.09 (0.04)	0.009	−0.03 (0.06)	0.58	0.11 (0.07)	<0.001	0.48 (0.19)	0.017
Handgrip Strength: age^°^	0.002 (0.001)	<0.001	0.001 (0.010)	0.047	0.001 (0.001)	0.022	0.001 (0.001)	<0.001	0.005 (0.001)	<0.001
Barthel: age^°^	−0.002 (0.001)	0.005	−0.001 (0.001)	0.005	−0.001 (0.001)	0.004	−0.001 (0.001)	0.065	−0.007 (0.002)	0.001
Lawton: age^°^	−0.008 (0.001)	<0.001	−0.002 (0.001)	<0.001	−0.001 (0.001)	0.299	−0.004 (0.001)	<0.001	−0.015 (0.003)	<0.001

° *Interaction; β, Beta coefficient; SE, standard error*.

**Figure 2:**
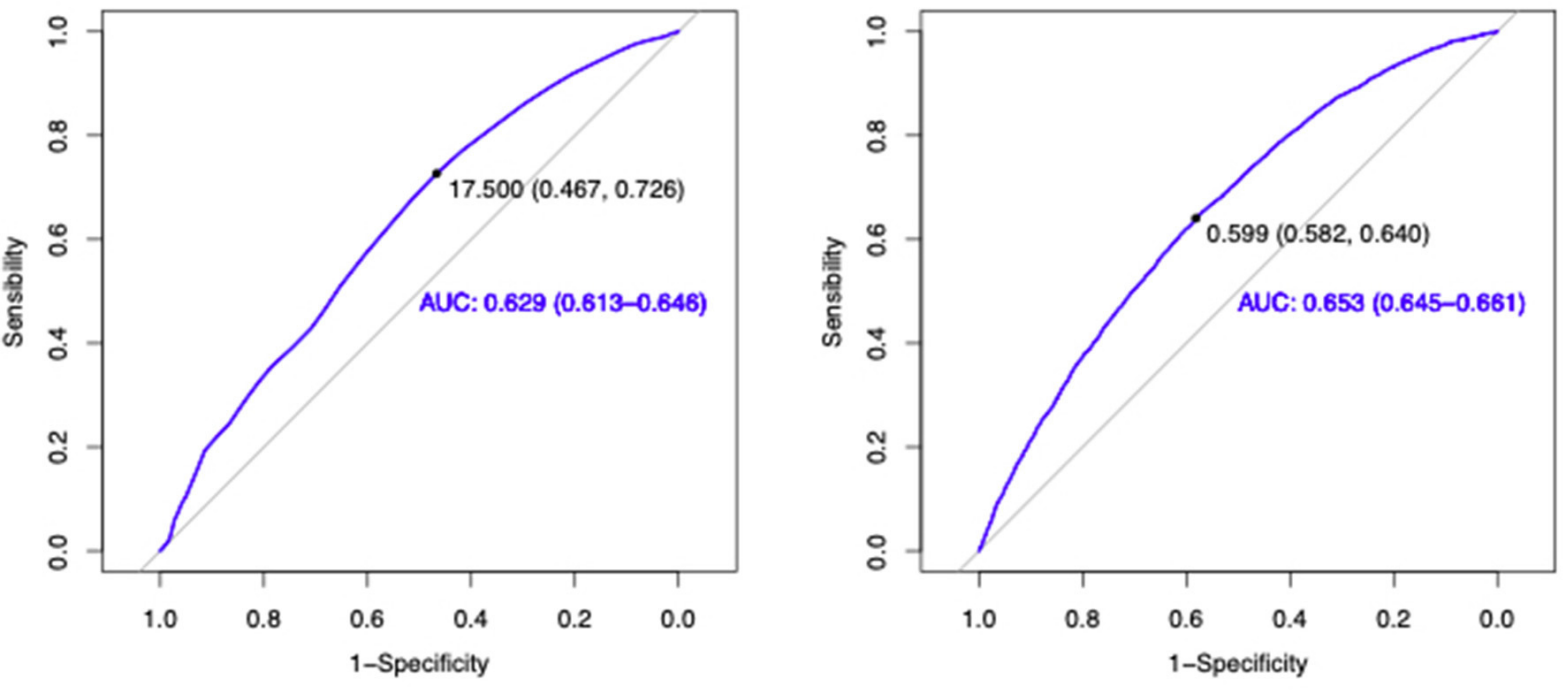
ROC curves. Receiver operating characteristic (ROC) curves defining cutoff points for HS **(A)** and GS **(B)** as markers for cognitive impairment. The dot represents the point with the largest AUC. Area under the curve: **(A)** 0.629 (CI 95%: 0.613–0.646), **(B)** 0.653 (CI 95%: 0.645–0.661). AUC, area under the curve; HS, handgrip strength; GS, gait speed.

Figure 2 shows the ROC curves that evaluated GS and HS as markers of cognitive impairment. Regarding GS, a cutoff point of 0.599 m/s was identified, and the curve had an AUC of 0.653 (95% CI: 0.645–0.661). Conversely, the curve for HS had an AUC of 0.629 (95% CI: 0.613–0.646), and the cutoff point that was established was17.50 kg. Supplementary Figure 1 shows the ROC curves for HS comparing cutoff points and AUC between women and men, with cutoff points of 16.5 kg in women, and 25.5 kg in men. The GS analysis by gender revealed a cutoff point of 0.59 m/s for females and 0.74 m/s for males (Supplementary Figure 2).

## Discussion

Results in this study support an association between muscular function and cognition. Motor function exhibited the largest association with the orientation domain, followed by language. Our findings complement preliminary reports showing associations among early motor function loss, dysexecutive symptoms, and impairments in the semantic memory (33).

The underlying neural mechanisms that may explain the motor-cognition relationship would be in the hippocampal place, grid, speed, and acceleration cells. Located in the entorhinal cortex, these cells play an important role in the spatial orientation and movement (34–36). In individuals without cognitive impairment, there is a reported correlation between a small volume of the left entorhinal cortex and muscular dysfunction in a dual-task gait assessment. The dysfunction consisted of gait slowing while performing a demanding cognitive task, such as counting backward, subtracting numbers, and naming animals (37). Previous reports suggest that executive functions are essential for gait control since gait requires the integration of sensory and perceptual information, a continuous updating of input, and quick adaptations of the gait pattern (38–40). Similarly, in a longitudinal study that performed a dual-task gait assessment, most of the GS variance was attributed to the level of executive attention (27.4%), and there was a link between orientation and attention (41).

Similarly, speech and language are among the most reliable markers distinguishing types of dementia that include motor dysfunction (42). Speech and language are also cognitive domains strongly associated with the supplementary motor area (SMA), a very important structure in motor execution. Alterations in the SMA and associated circuits (subcortical circuits, basal ganglia) may present clinically as alterations in language and motor performance (43). Muscle strength, specifically HS, can be an overall indicator of central nervous system integrity. Higher HS is associated with better performance on functional tasks, and it may indicate the ability to walk, rise from a chair, and hold small items such as a toothbrush or a comb (13).

Results in this study showed that for the MMSE domains, the determination coefficients (*r*^2^) are similar for GS and HS analyses. This suggests that GS and HS have a similar performance when assessing the correlation between motor function and cognitive state. It is relevant though, that this study did not find a strong correlation between GS and HS (Rho Spearman = 0.39). In addition, the AUC of GS and HS were significantly similar (0.65 and 0.63), which leads to the hypothesis that the two motor variables may be used independently to assess cognitive impairment with similar performance, especially when both variables do not present collinearity. Further studies may be needed to confirm this hypothesis. The GS has emerged as one of the motor domains strongly correlated with the incident dementia. Results in this study showed that GS and HS may be alternative parameters for the assessment of individuals at the risk of developing dementia, and also other geriatric syndromes such as frailty (44). Handgrip dynamometers are inexpensive, easily portable, non-invasive, and reliable. Their use does not require extensive training, and results are not biased by learning effects that can be seen in neuropsychological tests (13).

In line with our findings, previous reports have shown that poor physical performance is associated with cognitive decline. As well, GS and HS represent a core determinant of physical frailty and sarcopenia, both associated with cognitive impairment (45). It has recently been proposed that physical and cognitive decline can occur simultaneously and that they can share common etiologies (46). Hormonal levels and inflammatory biomarkers are thought to be implicated in cognitive dysfunction. For example, irisin myokine is expressed not only in the muscle, but also in the brain. It reduces neuroinflammation and post-ischemic oxidative stress, suggesting that this molecule may play an important role in neuroprotection and synaptic plasticity (47). Similarly, higher levels of proinflammatory cytokines, such as IL-6, were associated with greater cognitive decline and lower HS. Associations between impaired cognitive performance and poor physical performance in GS and balance suggest that abnormalities in the nervous system's processing speed might also be linked to changes in the cognitive function (13).

In this analysis, the accuracy of GS and HS as methods to identify cognitive impairment was 65 and 63 %, respectively. The cutoff point set for GS was 0.59 m/s and for HS 17.5 kg. Significant differences were seen between males and females in the HS ROC curve analysis, with cutoff points of 16.5 kg in women, and 25.5 kg in men. Similar values have been reported; in a cross-sectional study about the comprehensive geriatric assessment and GS, performed by the Chongqing Medical University, GS below 0.73 m/s had an AUC of 0.716 (48). Even so, a multivariate logistic regression reported by DeCock et al. (49) with a GS below 0.43 m/s predicted cognitive impairment with 88% accuracy. Also, a cross-sectional study using photocells and the Optogait System revealed that a gait step coefficient of variability above 3.9 s predicted the development of MCI with 85.2% accuracy (50). The model in this study did not include gait parameters such as cadence, step length, normalized speed, dual-task cost, swing time, and cycle time variability, all included in the aforementioned papers. Regarding HS, a cutoff of 20.65 kg was predicted with 71.2% accuracy functional decline in a hospitalized male cohort (51). In that cohort, more variables were included in the model, explaining its accuracy.

One of the main contributions of this study is that it proposes a non-cognitive method to identify older adults with cognitive impairment in a nationally representative sample of a middle-income country. It fills knowledge gaps in biomarkers in this field. This could, consequently, improve the prognosis of a population with access difficulties and give them an opportunity to receive specialized care. Given that there is no sufficient evidence using GS or HS to identify cognitive impairment, many recent publications suggest combining motor and cognitive measures to improve the classification of older adults at risk of dementia (52–55). So far, studies have reported the usefulness of these motor biomarkers, but there is a little evidence of which specific cognitive domains are related to the motor performance. This represents another contribution of this study, since measuring cognitive status with the MMSE allowed identification of the main cognitive domains associated with motor variables.

Around the world, and most particularly in middle-income countries, there is a pressing need to find low-cost, accurate, and accessible biomarkers to identify pre-clinical stages of dementia (56). The results of this study contribute to and enhance the opportunity for diagnosis in countries without access to expensive exams, such as Positron Emission Tomography (PET) and molecular biomarkers. This study's results also present an opportunity to establish preventive strategies based on risk assessments made with inexpensive and easy-to-apply measurements. This facilitates the access and training of health personnel, even in remote areas where populations have a high burden of disease and prevalence of dementia, but limited resources. Further research in this area may allow study findings to be generalized.

This study has some limitations. First, given that SABE is a cross-sectional study, this study could not establish causality. This demonstrates the importance of conducting longitudinal studies evaluating the predictive validity of HS and GS and standardizing the optimal cutoff for detecting individuals with impaired cognition. Second, this study did not include, in its ROC analysis, covariates such as age and schooling that may contribute to the discriminative power of the analysis. The statistic analysis did not include further covariates, since the intention when calculating cutoff points was for these to be generally applicable in a certain population. This possibility could be lost if multiple covariates (age, education, and other demographic variables) were taken into account. Even so, these covariates should be considered and included in future studies if they represent a significant difference between groups (cognitively normal and cognitively impaired).

It is also important to point out that the MMSE provides no information about executive functions, which represent a crucial cognitive domain in the evaluation of dementia in the elderly. Its deterioration is directly related to gait disturbances, as has been consistently found in the multiple studies (57, 58). Regardless, using the MMSE in this study's analysis allowed an evaluation of cognitive domains that had not been previously explored, and with which we found relationships with clinical relevance, as mentioned. Finally, the GS in the 3-meter test is not widely recommended, as results may underestimate a subject's speed (59). The authors do not consider that these biases study results, as it has been used in the previous research (9, 15). Furthermore, SABE Colombia includes the largest sample of Latin American older adults, providing good statistical power to this analysis, so long as the application of the results is carried out in populations with similar sociodemographic and anthropometric characteristics.

## Conclusion

Diminished GS and HS are associated with cognitive impairment, with the largest association in orientation and language domains. The GS and HS appear to be useful screening tools that can be used by any clinician to identify cognitive impairment. Both motor function tests share similar operational characteristics and can be used independently. These easy-to-use and accessible tools may be particularly helpful in low- and middle-income countries, reducing the costs associated with a full neuropsychologic assessment, PET imaging, or biomarkers, especially in the remote areas.

## Data Availability Statement

Publicly available datasets were analyzed in this study. This data can be found here: https://www.minsalud.gov.co/sites/rid/Lists/BibliotecaDigital/RIDE/VS/ED/GCFI/Resumen-Ejecutivo-Encuesta-SABE.pdf.

## Ethics Statement

Two universities involved in developing SABE Colombia (Universidad de Caldas, protocol ID CBCS-021-14, and Universidad del Valle, protocol IDs 09-014 and O11-015) reviewed and approved the study protocol. Written informed consent was obtained from each individual before inclusion and completion of the first examination (This included permission to use secondary data and blood samples). The protocol for a secondary analysis was approved by The Human Subjects Committee at the Pontificia Universidad Javeriana (Act ID 20/2017-2017/180,FM-CIE-0459-17).

## Author Contributions

EG-C provided the idea, coordinated the project, performed the statistical analysis, interpreted data, and discussed findings. FB-R performed the statistical analysis, analyzed data, and discussed findings. FR analyzed and interpreted data. IM, GG-O, and M-FA read and discussed findings. All authors read and approved the final manuscript.

## Funding

This study is part of a larger project funded by Colciencias and the Ministerio de Salud y la Protección Social, the Colombian Ministry of Health and Social Protection (The SABE Study, ID 2013, no. 764). Contract of financing RC No. 829 of 2018, Colciencias – Pontificia Universidad Javeriana.

## Conflict of Interest

The authors declare that the research was conducted in the absence of any commercial or financial relationships that could be construed as a potential conflict of interest.

## Publisher's Note

All claims expressed in this article are solely those of the authors and do not necessarily represent those of their affiliated organizations, or those of the publisher, the editors and the reviewers. Any product that may be evaluated in this article, or claim that may be made by its manufacturer, is not guaranteed or endorsed by the publisher.
