# Supplementary figures and images for: Muscular Function as an Alternative to Identify Cognitive Impairment: A Secondary Analysis From SABE Colombia
Source: Front Neurol. 2022 Feb 18;13:695253. doi: 10.3389/fneur.2022.695253 (PMC8896314; doi:10.3389/fneur.2022.695253)

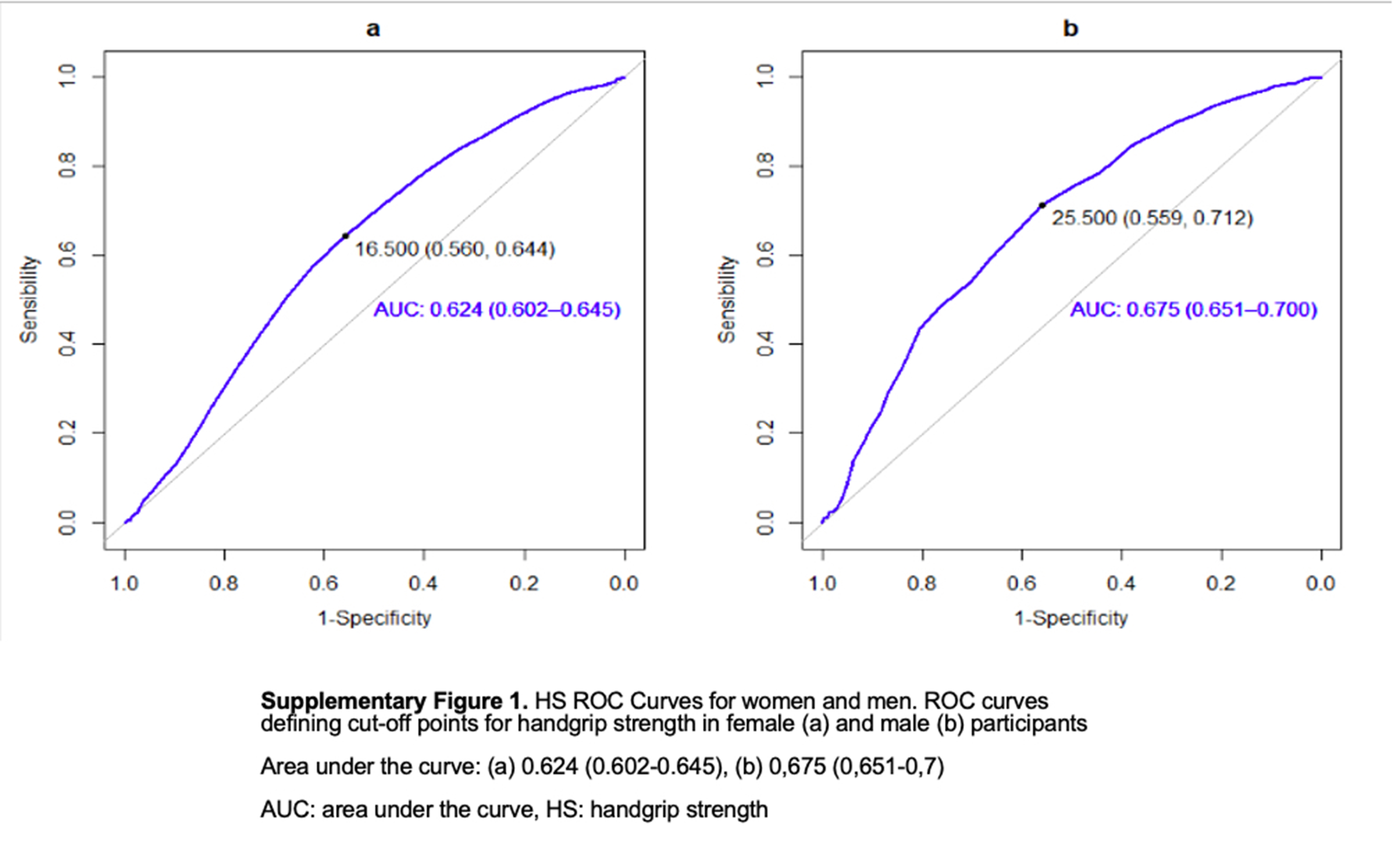

Supplement: Supplementary file 1 [file Image_1.jpeg]

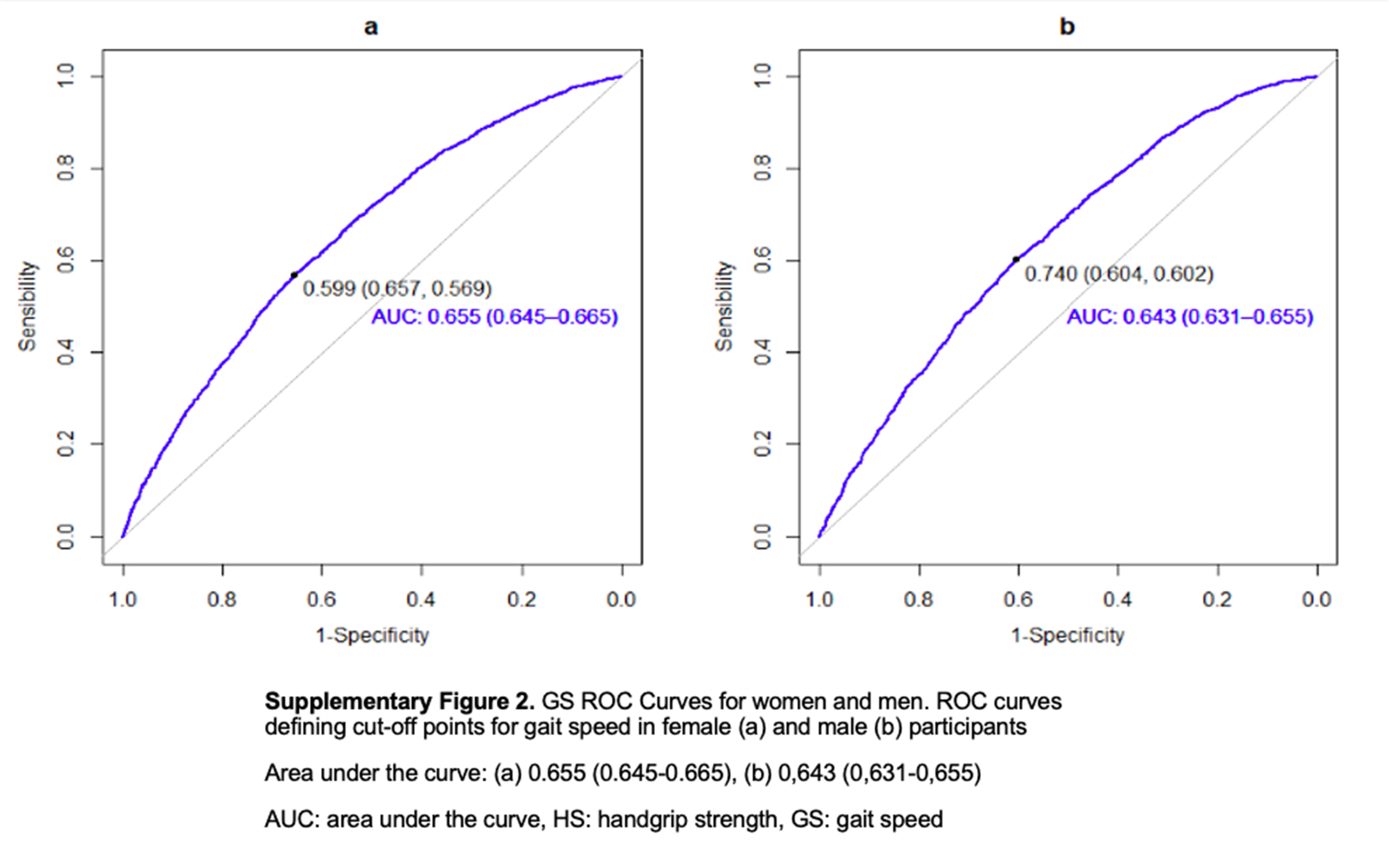

Supplement: Supplementary file 2 [file Image_2.jpeg]
